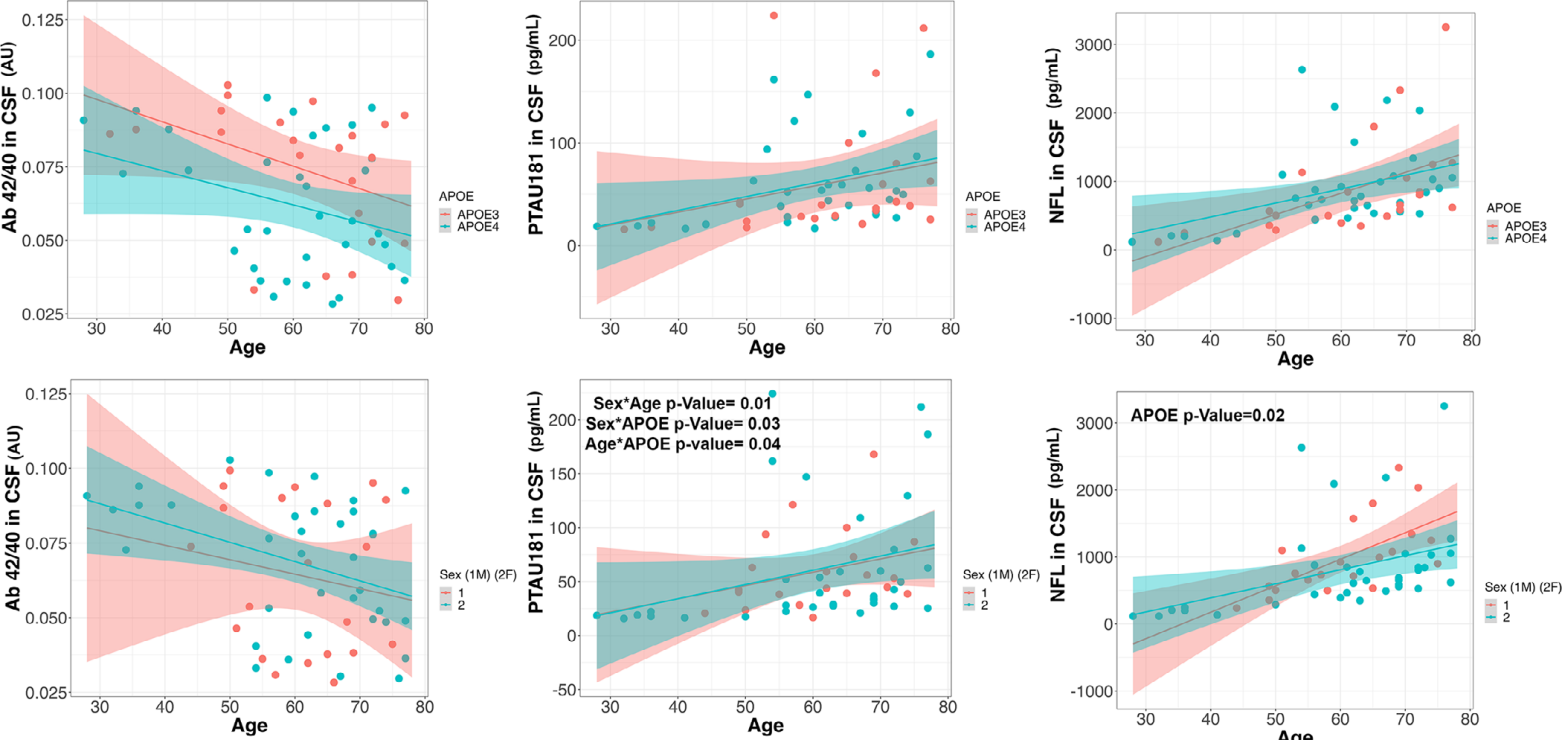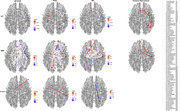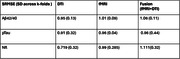# Brain Networks Associated with Alzheimer’s Disease Biomarkers

**DOI:** 10.1002/alz.089464

**Published:** 2025-01-09

**Authors:** Ali Mahzarnia, Jacques Andrew Stout, Robert J Anderson, Hae Sol Moon, Michael W Lutz, Ana‐Maria Staicu, Alexandra Badea

**Affiliations:** ^1^ Radiology Department at Duke University, Durham, NC USA; ^2^ Brain Imaging and Analysis Center, Duke University Medical Center, Durham, NC USA; ^3^ Radiology Department, Duke University Medical Center, Durham, NC USA; ^4^ Biomedical Engineering Department, Duke University, Durham, NC USA; ^5^ Duke Department of Neurology, Durham, NC USA; ^6^ Department of Statistics at North Carolina State University, Raleigh, NC USA

## Abstract

**Background:**

While we do not yet have the means to detect early Alzheimer’s disease (AD), studying subjects at risk conferred by the presence of the APOE4 allele, can provide useful information before clinical onset. We show that using symmetric bilinear regression with L1 penalty (SBL) of individual (DTI, fMRI) and fused connectomes, we can identify vulnerable regions changing in association with hallmark AD biomarkers measured in cerebrospinal fluid: amyloid beta Aβ42/40, phosphorylated tau (PTAU), and neurofibrillary light (NfL) as a proxy for neurodegeneration.

**Methods:**

We use structural connectomes derived from diffusion‐weighted MRI (DTI) and functional connectomes (fMRI) from 57 subjects, 45 normal controls and 12 cognitively impaired to predict CSF Aβ42/40, PTAU, and NfL to reflect neurodegeneration. To fuse connectomes, we first ranked them and then used the SNFtool. We run SBL for the three types of connectomes (DTI, fMRI, and fusion) and the three biomarkers. We compared the models using a k‐fold cross‐validations scheme and standardized root mean square errors (SRMSE) by dividing RMSE by the standard deviation of the biomarker outcome.

**Results:**

Linear modeling approaches showed that APOE is a significant factor with respect to NfL biomarker measures and that the interaction between APOE and age, APOE and sex, sex and age, were significant for PTAU (Figure 1). The SBL results showed that the best predictions were for NfL, PTAU and Aβ ratio based on DTI connectomes (Table 1.) The common sub‐network across biomarkers via DTI connectomes included the right inferior parietal, inferior temporal, superior parietal and caudal middle frontal, postcentral; and left superior frontal, right precentral, right superior frontal. The common connections across biomarkers via fMRI included the right putamen, left frontal pole and left accumbens, and right transversetemporal gyrus (Figure 2).

**Conclusions:**

DTI stood out as the best performer in predicting key biomarkers for AD (NfL, PTAU, Ab ratio). This research enhances our understanding of vulnerable brain networks, including in subjects at risk. Future work will explore strategies for data fusion to preserve common and unique contributions from multiple data sources.